# Pain in Covid Era

**DOI:** 10.3389/fphys.2021.624154

**Published:** 2021-02-02

**Authors:** Heloísa Alonso-Matielo, Victória Regina da Silva Oliveira, Victhor Teixeira de Oliveira, Camila Squarzoni Dale

**Affiliations:** Department of Anatomy, Laboratory of Neuromodulation and Experimental Pain, University of São Paulo, São Paulo, Brazil

**Keywords:** chronic pain, acute pain, comorbidity, SARS-CoV-2, pandemic (COVID19)

## Abstract

The COVID19 pandemic has impacted the lives and health of persons worldwide and although majority of COVID19 patients present with respiratory symptoms, pain emerges as an important feature of COVID19 infection. About 15–20% of patients progress to a severe condition that requires hospitalization. Although the disease was initially reported as a respiratory syndrome, other systems such as cardiovascular, renal, and nervous systems may be affected in the acute stages, increasing the need for continuous support to treat multiple sequelae caused by the disease. Due to the severity of the disease, damages found after discharge should also be considered. Providing multidisciplinary interventions promoting physical and psychological recovery in the first stages of hospitalization can minimize these damages. Cognitive, physical and psychological dysfunction reported by COVID19 patients after discharge can have profound effects on quality of life. Pain is usually part of this dysfunction, but it is still poorly understood how it affects survivors of COVID19 infections. There is limited information about the clinical characteristics, treatment and outcome of maintenance of pain in COVID19 patients. The purpose of this narrative review is to provide an overview of the implications of COVID19 on acute and chronic pain states.

## Introduction

The COVID19 pandemic has significantly impacted the lives and health of people worldwide, with potential for further effects in the future. The unprecedented changes which developed quickly due to the pandemic, have disrupted and affected everyone’s daily life, including those living with chronic pain (Puntillo et al., 2020). About 15–20% of patients infected with SARS-CoV-2 progress to a severe condition that requires hospitalization (Nanjayya, 2020). It is known that comorbidities such as diabetes, obesity, hypertension, cardiovascular diseases, immunodeficiency, among others, play an important role in the severity of COVID19, however, patients without comorbidities can also progress to severe cases requiring hospitalization. Although the disease was initially reported as a respiratory syndrome, other systems such as cardiovascular, renal and nervous systems may be affected in the acute stages, increasing the need for continuous support to treat multiple sequelae caused by the disease.

Chronic pain, as defined by the International Association for the Study of Pain (IASP), is a persistent or recurrent pain lasting more than 3 months or beyond the normal tissue healing (Puntillo et al., 2020). The overall prevalence of chronic pain in the general population is around 30% and its burden is huge in terms of personal and socioeconomic costs (Van Hecke et al., 2014). The SARS-CoV-2 pandemic has increased the risk of developing chronic pain due to viral infection, pain management or as a consequence of social isolation.

A consistent risk factor for the development of chronic pain is the occurrence of acute pain, it is worth considering how this is managed in hospitalized patients. Those who remember higher pain and distress during an ICU stay appear to be at greater risk of developing chronic pain after discharge (Nanjayya, 2020). It is likely that those who survive critical illnesses with COVID19 are at particular risk of developing chronic diseases such as chronic pain.

Cognitive, physical, and psychological dysfunction reported by COVID19 patients can have profound effects on quality of life (Nanjayya, 2020). Chronic pain is usually part of this dysfunction, but it is still poorly understood how it affects survivors of intensive care units (ICU). A main concern due to the severity of the disease, are damages found during and after hospital discharge. Providing multidisciplinary interventions promoting physical and psychological recovery in the first stages of hospitalization can minimize these damages (Eccleston et al., 2020).

Additionally, patients with chronic pain also have a higher risk of depression (Williams, 2003). Another concerning factor is that social isolation itself is a risk factor for the development of depressive symptoms. The present population suffering from chronic pain was seriously affected by social isolation, usually in-home confinement, as an important measure to mitigate the risk of COVID19 infection. Also, pain management services have been postponed or canceled, considerably diminishing the condition of the general population suffering from chronic pain (Eccleston et al., 2020; Shanthanna et al., 2020). In addition, physical well-being and mental health were deeply harmed, enhancing symptoms such as depression, anxiety, disruption of sleep, worsening pain status and resulting in a poorly quality of life. It is obvious that the relationship between chronic pain, COVID19-related mental disorders and those affected by social isolation can be drastic for chronic pain patients, with an additional impairment of their conditions and quality of life in general.

**FIGURE 1:**
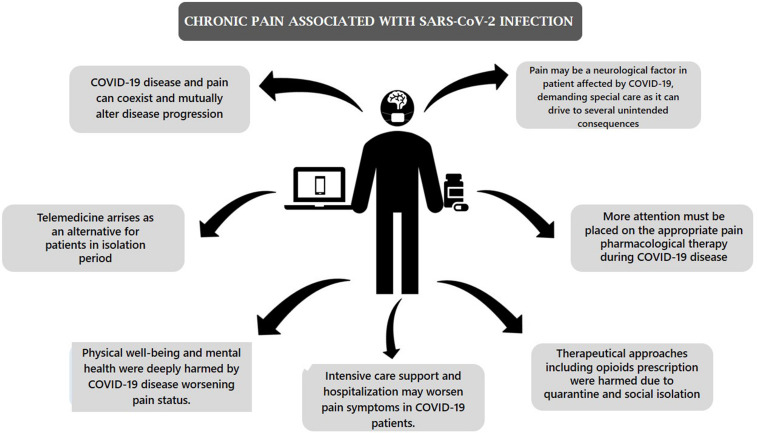
Summary of the pain scenario in COVID19 infection.

In this narrative review, we will examine the potential health consequences of COVID19 on chronic pain, by providing a summary and an argumentation of relevant published topics, in three different scenarios, including: (1) chronic pain as part of a post-viral syndrome or the result of viral-associated organ damage; (2) worsening of chronic pain due to exacerbation of preexisting pain physical or mental complaints; and (3) chronic pain by exacerbation of risk factors. Figure 1 summarizes the evaluate scenario.

## Acute Pain Associated With SARS-CoV-2 Infection Pathology

COVID19 is characterized as the novel coronavirus disease caused by the severe acute respiratory syndrome coronavirus 2 (SARS-CoV-2) that drives common or atypical symptoms such as fever, dry cough, fatigue, dyspnea, anosmia, diarrhea, and possibly resulting in patient’s death (Drożdżal et al., 2020; Shanthanna et al., 2020). Like most viral infections in which pain is a very common symptom, COVID19-infected patients commonly manifest headaches, sore throat, myalgia, arthralgia or peripheral neuralgias, not so different from what has been observed in many COVID19 patients, in which pain is also considered a major symptom (Drożdżal et al., 2020). However, different pain symptoms were linked to the current COVID19 infection as cofactors associated with the disease.

### Muscle Pain-Myalgia

Myalgia is one of the most common manifestations observed in COVID19 patients, appearing in nearly 36% of them (Li et al., 2020). In a first estimation it was pointed out that 14.8% of infections presented myalgia or arthralgia (Drożdżal et al., 2020; Nanjayya, 2020). In Hubei, China, one of the first affected areas, myalgia and fatigue were presented by 32.1% of patients (Liu et al., 2020). Increased release of cytokines such as IL-6, IL-10 and TNF-a (Drożdżal et al., 2020), as well as clinical laboratory markers of inflammation such as C-reactive protein (CRP), lactate dehydrogenase (LDH), and erythrocyte sedimentation rate (ESR) are elevated in patients with COVID19 with moderate to severe rates, thus suggesting the presence of generalized inflammatory response (Chen et al., 2020; Rodriguez-Morales et al., 2020), which could explain the presence of myalgia.

Weakness and hyporeflexia of the lower limbs have also been reported in association with the new coronavirus infection. Thus, not classical pain, but weakness of the lower limbs can be reported as suggestive of a motor peripheral neuropathy and can be present even before the appearance of first symptoms and clinical confirmation of COVID19 (Abdelnour et al., 2020).

### Abdominal Pain

Gastrointestinal symptoms are less common and more difficult to recognize as part of a COVID19 syndrome. Abdominal pain was reported following gastrointestinal manifestations, in 2–6% of infected general adults, teens or children (Oba et al., 2020). In most cases, pain was associated with the presence of diarrhea or anorexia (Dong et al., 2020). In this sense, Gahide et al. (2020) reported three cases of patients, clinically diagnosed with COVID19, who presented acute abdominal pain and lung injuries, without other major respiratory symptoms or fever at the time of hospitalization, indicating that abdominal pain is a relevant occurrence for COVID19 diagnosis and treatment. Also, Poggiali et al. (2020) reported 10 cases of patients presenting fever and flu like symptoms in the previous 5–10 days, with general malaise, decreased appetite, abdominal pain and diarrhea. Angiotensin-converting enzyme 2 (ACE2) highly expressed in the human small intestine, is the main described cell receptor for the novel coronavirus (Su et al., 2020). Diarrhea occurs secondary to the interaction between ACE2 receptor and SARS-CoV-2. Recent studies show that SARS-CoV-2 RNA was detected in stool samples, confirming fecal-oral transmission (Poggiali et al., 2020). These data reinforce the presence of abdominal pain as a differential symptom in COVID19 infection.

Generalized inflammation in gastrointestinal system (gastroenteritis) was suggested as possible mechanism associated to abdominal pain in COVID19 patients, however, it should be noted that patients diagnosed with inflammatory bowel disease or chronic liver disease do not present elevated risk for COVID19 compared to the general population (Oba et al., 2020).

### Neurological Manifestations

Central pain has also been suggested as a possible COVID19 neurological manifestation (Drożdżal et al., 2020), thus increasing the concern for the development of pain accompanied by other coronavirus sequels. Headache is highly prevalent in infected individuals with COVID19, and in some surveys up to 90.5% of infected patients reported headaches as a first symptom (26%) or a symptom that appeared up to 48 h (62.5%) after admission at the emergency service (Lechien et al., 2020; Trigo et al., 2020). Headache was accompanied by anosmia, arthralgia, cough, light headedness, and myalgia (Trigo et al., 2020). Generalized inflammation, release and increase of cytokines, injury to endothelial vessels, macrophage activation, and increased glial activation are some of the mechanisms suggested for pain (Drożdżal et al., 2020; Kanberg et al., 2020; Trigo et al., 2020). Increased expression of ACE2 receptors in spinal neurons would play a role in pain sensibility, and therefore, central pain. However, further investigation are still necessary to demonstrate the exact mechanisms leading to central pain as well as causes and correlation of headache with COVID19 severity (Su et al., 2020; Trigo et al., 2020).

## Chronic Pain and COVID-19

### Worsening of Chronic Pain Due to Exacerbation of Preexisting Pain, Physical, or Mental Complaints

Of the people affected with chronic pain in the general population, 85% suffer from severe depression (Williams, 2003). Chronic pain patients that do not receive adequate treatment for their pain condition, present drastic levels of depression (Choinière et al., 2010). A specific serotonergic pathway from the dorsal raphe nucleus to the lateral habenula, via the central amygdala was identified as a key neural circuit governing depressive symptoms in chronic pain (Tappe-Theodor and Kuner, 2019; Zhou et al., 2019). Chronic pain may induce depression and vice versa, depression may cause abnormal pain perception and modulation, with increased risk of developing chronic pain (Currie and Wang, 2005; Tappe-Theodor and Kuner, 2019). High levels of anxiety or presence of anxiety disorder have been observed in more than 50% of individuals with chronic pain. Neuroimaging studies suggest that overlapping brain areas, such as thalamus, prefrontal cortex, and anterior cingulate cortex are activated by both chronic pain and anxiety (Hsieh et al., 1996; Davidson et al., 1999).

Almost 60% of the people affected by COVID19 have been affected in one or more social and daily activities such as; sleep, diet, and exercise. The most frequently reported problem was pain/discomfort (19.0%) and anxiety/depression (17.6%). Logistic regression models demonstrate that the risk of pain/discomfort and anxiety/depression triggering factors related to mental disorders have significantly raised in particular population groups (Lei et al., 2020; Majumdar et al., 2020; Ping et al., 2020). Among them elderly and people affected by chronic diseases, lower income, and those concerned with acquiring the COVID19 which also develop stress, anxiety and depression acquire higher risk of pain (Lei et al., 2020; Majumdar et al., 2020; Ping et al., 2020).

Individuals with chronic diseases report more psychological symptoms than the rest of the population (Ozamiz-Etxebarria et al., 2020) and social isolation due to COVID19 pandemic intensified those symptoms. Thus, social isolation itself added to all reported consequences of COVID19 outbreak, are considered risk factors in respect to the development or even decreasing mental health and exacerbate pre-existing conditions which, in turn, could adversely impact pain-related treatment outcomes (Cohen et al., 2020). Moreover, the number of people suffering from mental illness after a major event is often greater and its effects may last longer (Ping et al., 2020), especially in people with chronic disease, elderly and lower income population.

### Chronic Pain by Exacerbation of Risk Factors

Chronic pain is a highly prevalent condition which has high cost while impairing quality of life and implying personal disabilities requiring health, economic and social efforts (Mills et al., 2019). Of notice, chronic pain occurs significantly more in the elderly population, already reported as higher risk for developing severe COVID19 (Chen et al., 2020; Shanthanna et al., 2020).

Patients are more likely to develop pain or discomfort and anxiety/depression, while worrying about being infected with SARS-CoV-2 and developing severe symptoms of COVID19 (Ping et al., 2020). Data peaks in the elderly, people with chronic diseases and individuals with low incomes or worried about get COVID19 during the COVID19 pandemic (Ping et al., 2020). This data reinforces the urgency to observe, diagnose and address painful symptoms during COVID19 outbreak.

During the novel COVID19 pandemic, chronic neuropathic pain, neck and back pain, orofacial pain, or headaches, besides being consequences of major SARS-CoV-2 infection, may also be increased by intensive care support during hospitalization, or directly influenced by the loss of health care facilities which stopped their activities following governmental orientation. Unpleasant sensations, discomfort and continuous ongoing pain are marked outcomes in patients hospitalized in ICUs that require interventive life supporting procedures such as sedation and mechanical ventilation (Drożdżal et al., 2020). Also, under ICU treatment conditions, COVID19 patients frequently are unable to personally report scales of pain, increasing the need for caring for patients pain as a potential underestimated sequel and suggestive for the use of others assessment tools (Drożdżal et al., 2020), as shown previously for assessment of pain scores in intubated patients (Critical Care Pain Observation Tool–CPOT) (Kotfis et al., 2018) or patients under sedation (Behavioral Pain Scale–BPS) (Payen et al., 2001) allowing to start pain monitoring still during hospitalization as a way to prevent further aggravation on pain reports after COVID19 recovery.

### Treating Chronic Pain on COVID19 Infections

Patients suffering any kind of pain during COVID19 pandemic demand special attention, as it can be driven by several neurological factors (Drożdżal et al., 2020) and may potentially be aggravated by pain or lead to chronic pain, a condition in coronavirus survivors that will require professional assistance for adequate analgesia and pain relief (Su et al., 2020). Thus, healthcare professionals urge to ensure continued care of acute and chronic pain in patients.

Facing this isolation period, individuals with higher pain burden (including chronic pain) are more likely to experience higher incidence of COVID19 infections. Thus, with the disruption of their usual healthcare access, the consequences of abruptly interrupted/altered healthcare treatment will diminish patients conditions (Eccleston et al., 2020). The risks of harm from under treatment can be aggravated further by inadequate treatment (Eccleston et al., 2020). Despite the fact that pharmacological therapies for pain management and related syndromes tend to be ineffective, negatively affecting the quality of life of individuals (Shamji et al., 2017; Campos et al., 2020), opioids and non-steroidal anti-inflammatory drugs are commonly used in the treatment of acute and chronic pain, even considering their adverse effects, tolerance and potential for addiction (Busse et al., 2018; Szok et al., 2019; Campos et al., 2020). Thus, the impact of the cessation of pain treatment caused by the COVID19 pandemic can lead to several unintended consequences, such as increased pain, reduced function, increased reliance on opioid medications and potential increased morbidity (Deer et al., 2020; El-Tallawy et al., 2020). The effect of opioids on the immune system seems to be complex and have been linked to infection in individuals on chronic opioid therapy. Therefore, its use by immunocompromised patients should be cautious and limited (Plein and Rittner, 2018; Cohen et al., 2020; El-Tallawy et al., 2020). With quarantine and social distancing in COVID19 pandemic there was a worsening of opioid use disorders, hence more attention must be placed on the appropriate prescription of these medications. Non-opioid strategies were suggested (e.g., using clonidine) to prevent opioid withdrawal and in last case, for patients at risk of opioid withdrawal, an in-person visit should be scheduled (El-Tallawy et al., 2020).

Notwithstanding, chronic pain management during COVID19 pandemic must be considered as important as the need of continuous supportive care, to recognize that health professionals worldwide could lack guidelines to deal remotely with patient’s pain therapeutics, from diagnosis to analgesics prescription and readily a new way of work, had to be carried out, once health facilities have paused activities and telemedicine was adopted (Shanthanna et al., 2020), a fact that may also influence the incidence of chronic pain.

## Discussion and Conclusion

In the present COVID19 pandemic many unknown factors still have to be identified, in order to understand the relationship of pain in COVID19-patients. Mechanisms for shutting down pain triggered by the virus have also been recently suggested and should be further explored (Moutal et al., 2020) since it may help in explaining the variability of pain symptoms. Despite the large number of COVID19 patients and manuscripts, there is still a lack of epidemiological studies focusing on pain symptoms. These studies should have more comparable criteria for selecting subjects in order to obtain a more representative picture of pain symptoms in COVID19. To better comprehend the mechanisms involved in the disease and the role of pain in the development of the infectious condition, pain should be analyzed as a consequence of the disease. Admitting that social isolation play a very important role in worsening pain cases is an extreme need for treatment and improvement in the quality of life of patients with chronic pain and other psychiatric comorbidities. Epidemiological data should be used to assist future health policies that seek to reduce the magnitude of future epidemics and their many consequences for chronic pain. The recognition that COVID19 induces chronic pain and exacerbates pre-existing chronic pain will be of utmost importance for a better understanding of the disease. In addition, immediate and targeted treatment as well as strategies to reduce the potential impact of chronic pain should be strongly encouraged.

## Author Contributions

All authors participated in the writing of this manuscript. All authors contributed to the article and approved the submitted version.

## Conflict of Interest

The authors declare that the research was conducted in the absence of any commercial or financial relationships that could be construed as a potential conflict of interest.
